# Novel Internet of Things based approach toward diabetes prediction using deep learning models

**DOI:** 10.3389/fpubh.2022.914106

**Published:** 2022-08-24

**Authors:** Anum Naseem, Raja Habib, Tabbasum Naz, Muhammad Atif, Muhammad Arif, Samia Allaoua Chelloug

**Affiliations:** ^1^Faculty of Computer Sciences, Ibadat International University, Islamabad, Pakistan; ^2^CMAC Future Manufacturing Research Hub, University of Strathclyde, Glasgow, United Kingdom; ^3^Department of Computer Science and IT, The University of Lahore, Lahore, Pakistan; ^4^Department of Information Technology, College of Computer and Information Sciences, Princess Nourah bint Abdulrahman University, Riyadh, Saudi Arabia

**Keywords:** deep learning, medical-Internet of Things, machine learning, diabetes, RNN, CNN

## Abstract

The integration of the Internet of Things with machine learning in different disciplines has benefited from recent technological advancements. In medical IoT, the fusion of these two disciplines can be extremely beneficial as it allows the creation of a receptive and interconnected environment and offers a variety of services to medical professionals and patients. Doctors can make early decisions to save a patient's life when disease forecasts are made early. IoT sensor captures the data from the patients, and machine learning techniques are used to analyze the data and predict the presence of the fatal disease i.e., diabetes. The goal of this research is to make a smart patient's health monitoring system based on machine learning that helps to detect the presence of a chronic disease in patient early and accurately. For the implementation, the diabetic dataset has been used. In order to detect the presence of the fatal disease, six different machine learning techniques are used i.e., Support Vector Machine (SVM), Logistic Regression, Artificial Neural Network (ANN), Convolutional Neural Network (CNN), Recurrent Neural Network (RNN), and Long Short-Term Memory (LSTM). The performance of the proposed model is evaluated by using four evaluation metrics i.e., accuracy, precision, recall, and F1-Score. The RNN outperformed remaining algorithms in terms of accuracy (81%), precision (75%), and F1-Score (65%). However, the recall (56%) for ANN was higher as compared to SVM and logistic regression, CNN, RNN, and LSTM. With the help of this proposed patient's health monitoring system, doctors will be able to diagnose the presence of the disease earlier.

## 1. Introduction

The Internet of Things (IoT) (1–4) is a network environment in which each connected unit can interact with several other components within the network to send vital data for precise and real-time decision making. Today, IoT is a significant milestone in the wave of digital growth. As a result, IoT plays a vital role in critical scenarios such as medical applications. IoT efficiently serves as a platform for the development of smart medical-care systems (5). IoT is a turning point in the history of technological growth, particularly for millennials, who have only lived through the post-globalization era of the human history. Machine learning is a field of artificial intelligence (6–8). Since medical data has become digitized, machine learning plays an essential role in the detection of numerous diseases. In the last decade, researchers have looked into a variety of machine learning approaches in the health-care industry (9–11). Recent technological advancements have resulted in the successful integration of the Internet of Things and machine learning in a variety of fields. In the clinical setting, the coupling of these two fields i.e., machine learning and IoT can greatly aid in the creation of a receptive and interconnected environment and as a result, healthcare professionals and patients will benefit from a variety of services (5, 12, 13). While IoT is concerned with the extraction of information and resources, machine learning is concerned with data analysis, data expansion, learning, and taking decisions based on input data. IoT's main goal is to make the world around us smarter by delivering the necessary data on a historical or real-time basis and by implementing machine learning and artificial technologies make smart decisions. In today's world, many things contribute to an inappropriate way of life, including irregular eating habits, a lack of nutrition, pollutants, lack of sufficient exercise, unending work, impatience, and elevated stress levels, all of which contribute to worsening of human health. An inactive lifestyle affects up to 40 percent of youngsters, middle-aged people, and career women in various nations. Because of our hectic daily schedules, we barely have time to focus on our health, leading to a variety of health problems (14). Furthermore, it would be challenging for a doctor to keep a constant eye on the patient. Individual patients may find it challenging to maintain state of their health condition and seek guidance from their doctors. Chronic diseases are defined as those that last a long time and necessitate long-term therapy. Patients with severe conditions are frequently admitted to the hospital for extended periods of time to be monitored on a regular basis. Heart disease, cancer, and diabetes are examples of common chronic diseases (15). Diabetic disease is currently exceedingly serious, as it kills a large number of individuals each year. As a result, in order to live a normal life, the diabetic patient must be monitored on regular basis. Diabetes is a chronic condition (16–18) caused by pancreatic dysfunction, which happens when the organ does not make enough insulin or when the body does not use it adequately. Many organs, including the eyes, neurons, and internal organs, can become defective or worsen as a result of high or low blood sugar levels. Therefore, to prevent the diabetic patient's health from deteriorating, continual and daily monitoring is necessary. Current medical-care software platforms, such as health management systems, patient monitoring systems are insufficient in assisting treatment with necessary information. The objective of this research is to make a patient health monitoring system especially for those who are suffering from chronic diseases like diabetes. It is a novel application designed by using various machine learning models. In this application, the patient's data is extracted by using IoT wearable technology. The data collected from a variety of medical wearables is fed into this monitoring system. Afterwards, this application uses machine learning algorithms on raw data to evaluate the patient's health situation in order to make appropriate decisions and diagnoses. This application predicts the patient's state and in case of emergency, alarming notification is sent to the doctor and guardians to provide immediate surveillance. This paper's key responsibilities are as follows:

1. In an IoT platform, providing a healthcare model based on machine learning and deep learning.
2. Checking patient's health state by measuring two types of health condition metrics in real time: biological and behavioral changes.
3. To produce the best forecast, the patient's health status is categorized by using SVM, logistic regression and ANN, CNN, RNN, and LSTM.

The remainder of this article is structured in the following manner: The Section 2 looks into the work that has been done in the past. The methodology for our proposed application model is covered in Section 3. In Section 4, we present our experimental findings, and in Section 5, we wrap up this article and discuss the future work.

## 2. Related work

A variety of health systems is invented to aid in rapid diagnosis and constant monitoring of a patient's condition. Different techniques have been used in the suggested systems to monitor the patient's health. IoT applications are playing vital role in medical world. In latest years, a lot of studies on IoT-based medical systems have been published. In one approach (19), researchers used machine learning and IoT to detect stress beforehand by using patient's heartbeat rate. To find out the heart rate of patient, a pulse sensor is used. The reading of pulse sensor is sent to the node MCU which is Wi-Fi module along with 32-bit microprocessor. Two algorithms for classification are being used. VF - 15 algorithm is a feature interval-based classifier, which creates classification intervals during training and uses it to test the classifier. This classifier gives an accuracy of 62%. The second classifier is Naive Bayes approach which is a Bayesian classification algorithm and gives 50% of accuracy while testing. They applied SVM and Logistic Regression which show considerable improvement over VF - 15 and Naive Bayes. Limitation of this paper is inadequate data, as any machine learning algorithm can only give correct readings/predictions if it is applied on reliable data. In (20), the proposed model consists of three layers i.e., IoT layer, cloud layer, and student's health monitoring layer. In the first phase, patient's data are obtained through medical devices and sensors. In the second phase, data mining techniques are performed on patient's data. In the third step, parents will be given information about student health if needed. To evaluate the proposed model, four data mining algorithms i.e., decision tree (DT), random forest (RF), SVM, and multilayer perceptron (MLP) are utilized in this paper. The future work of this study is to suggest an edge-based data processing system to bring computation and data storage closer to the patient's location to improve emergency services response time and save bandwidth in the system (20). In this research (21), the authors have investigated the effects of stress and for this purpose they used the sensor data from the accelerometer and gyroscope on smart phones. The authors monitored the writing behavior of 46 participants on the touchscreen panel of smart phones. The collected dataset contained 112 different features. In order to rank these features, Gain Ratio feature selection algorithm was applied. The authors applied a range of classification algorithms on these features such as Decision Trees, Bayesian Networks and k-Nearest Neighbor methods. The authors observed that the accuracy of these classifiers was 74.26, 67.86, and 87.56% respectively (21). In paper (22) discusses applicability of Intelligent IoT based on Collaborative Machine Learning in healthcare and medicine by presenting a holistic multi-layer architecture. The feasibility of such architecture is investigated by a case study, ECG-based arrhythmia detection, based on deep learning and Convolutional Neural Network (CNN) methods. This paper introduces an IoT application framework E-Healthcare Monitoring System (EHMS) integrated with Machine learning (ML) techniques to design an advanced automation system. In (23), for experimental purposes, they have used a diabetic data set. The dataset was passed through a machine learning algorithm called SVM which has achieved an accurate report. As future work, more Machine Learning algorithms can be applied to improve efficiency of the proposed system. The work presented in (12) proposes a health care system based on random forest classifier and IoT. The proposed system will improve interactivity between patients and doctors. Experimental results are conducted using various datasets related to different diseases. The machine learning techniques that have been employed in this work are K-NN, Support Vector Machine, Decision Trees, Random Forest, and MLP. Maximum accuracy of 97.26% has been achieved on Dermatology dataset using Random Forest machine learning technique. As a future work, the proposed system can be extended to other applications such as earth observations, weather forecasting etc. In (24), the authors have proposed a system to monitor the real time health parameters in order to monitor health of the soldiers in real time who become lost and get injured in the battlefield. The authors used various sensors for data acquisition. In order to transmit this data from sensors to the cloud, they used the network infrastructure like LoRaWAN and ZigBee. In order to analyze this data and predict warzone environment, the authors used K-Means Clustering machine learning algorithm. Although K-Mean Clustering produced useful predictions, density-based clustering algorithms such as DBSCAN could improve the performance since it can discover clusters of arbitrary shapes as well. In this paper (25), the authors have proposed a fuzzy discernibility matrix (FDM) based feature selection technique using a parameter K for Motor Imagery EEG Signal Classification. Based on the accuracies produced by the Support Vector Machine (SVM) and Ensemble variants of classifiers, the authors established that the proposed approach outperformed the state-of-the-art approaches. The results could be further improved by using an adaptive value of K. Machine learning (ML) is an influential technology for extracting information from IoT data. Healthcare has adopted IoT and machine learning so that computers can automatically create medical records, anticipate illness diagnoses, and, most critically, keep track of patients in real time. Understanding the various machine learning methods used to analyze IoT data in the healthcare sector is critical. Different researches provide a complete overview of existing machine learning (ML) algorithms and their use in IoT medical data (26, 27). The Internet of Things (IoT) offers potential solutions for reducing the pressure on healthcare systems. For example, RFID technologies are utilized in medical facilities to reduce medical costs and improve healthcare provision. Doctors can quickly monitor patients' cardiac impulses through healthcare monitoring schemes, which helps them provide an appropriate diagnosis (28). To evaluate the ECG signal, authors in (29) proposed an IoT-enabled ECG monitoring system. Firstly, the raw ECG signal's statistical features are calculated. Then for acquiring the dynamic aspects of the ECG data, the Pan Tompkins QRS detection algorithm is used to evaluate the signal. The system is employed to obtain RR intervals from ECG signals to extract heart rate variability properties. The cardiac arrhythmia disorder is then diagnosed using statistical and dynamic variables added to the classification method For effective encoding of the ECG signal in time-frequency space, a discrete orthogonal transform employing discrete cosine transform was developed in (30). In this paper feature extraction was offered to reduce the feature set, and support vector machine with particle swarm optimization as a tuning parameter was proposed for improving the accuracy of ECG signal classification. In (31), an artificial neural network classifier with Bayesian normalization back propagation was developed to automatically categorize the rhythms of ECG signals and distinguish resuscitation cardiac rhythms. To enhance the classifier's accuracy, feature selection was implemented using a wrapper-based strategy.

Most of the aforementioned approaches have proposed techniques to predict diabetes by way of machine learning algorithms such as decision trees, random forests, and SVM etc. These approaches have been evaluated using the measures like accuracy, F1-score, and recall, etc. Some of the approaches used a hybrid of machine learning and Internet of Things in order to make predictions about diabetes in patients.

In this research, we have proposed a hybrid approach that is based on Internet of Things and deep learning neural networks in order to make more accurate and precise predictions about diabetes. By virtue of IOT based smart devices, patients health status can be gathered in real time. So this makes the data more dependable and accurate. This research focuses on using the deep learning neural networks owing to their supremacy in terms of accuracy especially when trained with huge amount of data.

## 3. Proposed methodology

Our suggested monitoring system is an IoT application which is used to monitor patient's medical state and periodic check-ups for a variety of chronic illnesses. In proposed system, IoT wearable technology is used to gather data from patients in a variety of medical centers, residences, and workplaces. IoT wearable diagnostics equipment can detect irregular symptoms (such as increased heart rate) that may have gone unnoticed during doctor visits. When a patient's symptoms are so infrequent, a doctor may advise wearing wearables to keep track of them. IoT based smart devices enhance patient outcomes by enabling fact-based disease diagnosis. Different IoT wearables are used for early illness detection e.g., body temperature sensors, sweat sensors, heart rate monitors, glucose monitors, and echocardiogram monitors. An IoT sensor that identifies a patient's unusually low heart rate may send out a warning, allowing medical personnel to intervene. When an IoT device captures patient data, it sends it to a software program where ML algorithms are used to analyze data and make treatment recommendations or alarms. Machine learning classification algorithms are used to generate the training models from the data gathered, which is instantly saved in the local server. Prediction, review analysis, decision making, and data visualization are all done with the data collected from patients. This can be discussed by doctor as well as patient.Our proposed monitoring system employs a few Internet of Things (IoT) smart wearable devices that transmit real-time data from the human body. Temperature, pulse rate, blood pressure, and diabetes sensors based on eye lances are all compatible. By employing the Internet of Things (IoT) technology and Machine Learning approaches, this system provides an innovative application model that provides a better approach and considerable enhancement for many specialized health services.

**Figure 1:**
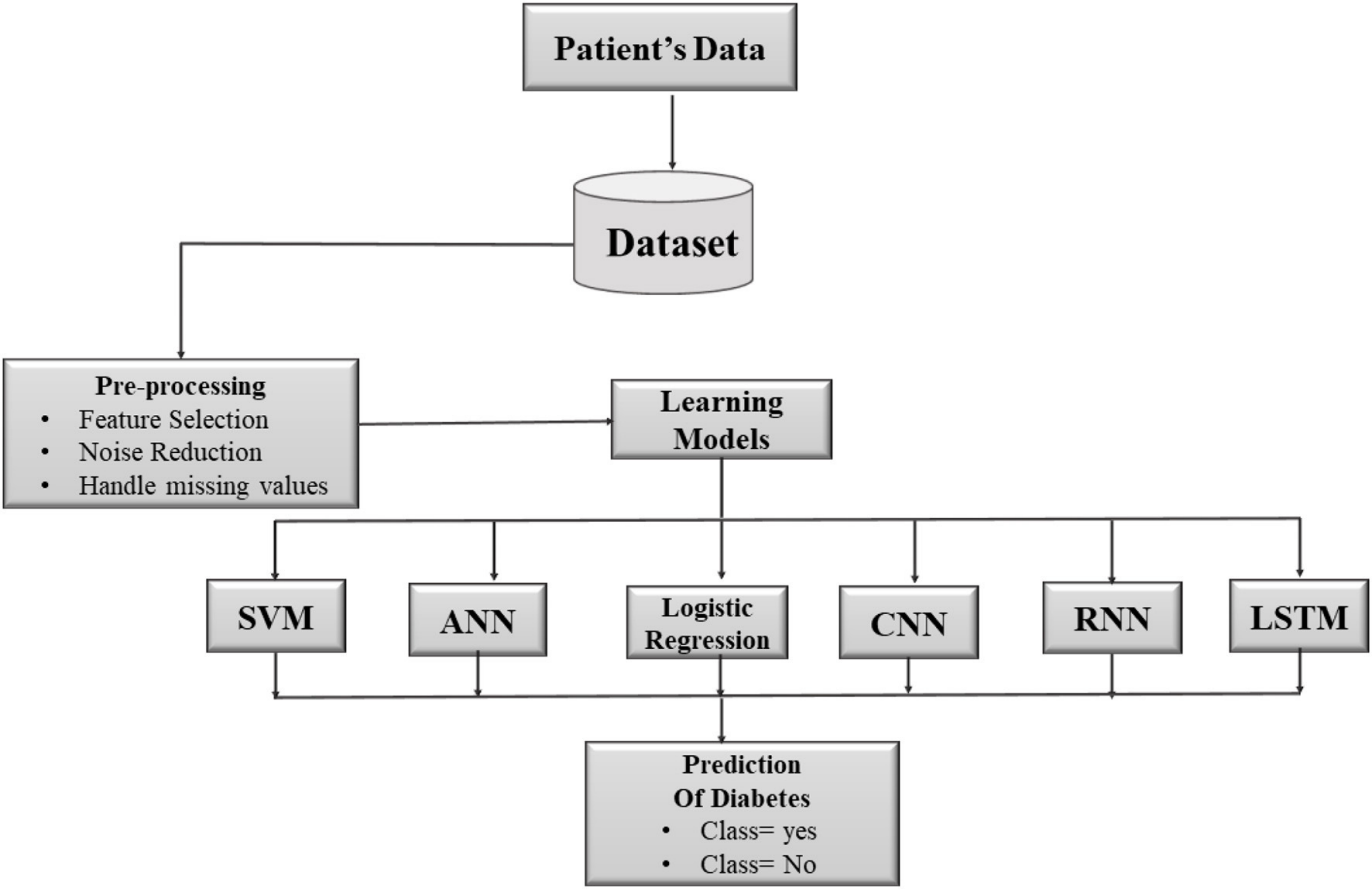
Architecture of smart patient's health monitoring system.

Figure 1 represents the overall working of the proposed model. First of all, by using IoT sensors patient's data is collected. Doctors evaluate the information they acquire in order to forecast sickness. Different machine learning classification methods were employed in the proposed model to classify the obtained data in order to distinguish between healthy and unhealthy patients. Machine learning classifiers are able to forecast the disease in a timely and accurate manner. We have used six different machine learning classifiers i.e., logistic regression, Support vector machine (SVM), and artificial neural network (ANN), Convolutional Neural Network (CNN), recurrent neural network (RNN), and Long Short-Term Memory (LSTM). The implementation of a suggested framework, there are three stages to the project. The first stage is data collecting; the second is data pre-processing and computing; and the third is data transparency to doctors. Patient data is collected from a variety of sources, including the patient's home, hospital, or clinic, as well as distant data. The patient data is collected in real-time using a variety of sensors and connected devices. The collected data is analyzed and validated for incomplete data during pre-processing. After the data has been pre-processed, it is transferred to the server for analysis. Six machine learning algorithms i.e., logistic regression, SVM, ANN, CNN, RNN, and LSTM are used to compute and evaluate the data. In order to implement the learning models, we divided our data into 80:20 training and testing datasets.

### 3.1. Support vector machine

SVM is the supervised learning approach (32). SVM is based on statistical learning theory. For binary classification and multi-class issues, the SVM method is used. The SVM approach creates massive hyperplanes in multi-dimensional area by optimizing the distance among data points and constructing a hyperplane with support vectors (33).

### 3.2. Logistic regression

Logistic regression (34) is a classification procedure that assigns facts to a defined specific class. It's a probability-based predictive analytic algorithm. Logistic regression may be used to categorize findings based on many forms of data and can quickly identify the most efficient variables for classification.

### 3.3. Artificial neural network (ANN)

Another most famous machine learning approach is artificial neural network (ANN) (35, 36), which is based on feed-forward neural networks. The input, hidden, and output layers are the three layers of an ANN. The input layer receives attribute input and uses hidden processing to transform the data into output for the output layer. The output layer transmits the output back to the hidden layer for more processing until the desired result is obtained. The modification is made during the training procedure. With the help of the hidden layer, the output layer decreases the output error. After the result is computed, the result is sent to the doctor in order to provide immediate surveillance to the patient in case diabetes is diagnosed.

### 3.4. Convolutional neural network (CNN)

CNN (37, 38) is a type of feedforward neural network designed to deal with deep network configurations. In CNN design, there are three levels layers: (1) Convolution layer: Translation inversion is provided by a convolutional layer. Because the convolution kernels operate on every portion of the sensor observation tensor, they are generally looking for the same feature throughout the tensor. The edge characteristics are extracted by the shallower convolution layer. The deeper convolution layers, on the other hand, retrieve the prospective features. (2) Pooling layer: After a convolution layer, a pooling layer is used to down-sample the feature maps created by the convolution layer. (3) Fully connected neural network layer: The fully connected layer is built in the same way as a regular neural network. Each hidden or output layer neuron is coupled to every input unit (that is, the outputs of the last pooling layer). The categorization results are generated by this layer.

### 3.5. Recurrent neural network (RNN)

RNN (39, 40) is made up of several layers with feedback loops that can propagate information from the past to the present. An RNN is made up of loops that allow the information to survive. The RNN's hidden layers serve as data storage, similar to computer memory. RNNs are a type of powerful DNN that deals with sequence data using its internal memory and loops.

### 3.6. Long short-term memory (LSTM)

The LSTM (41, 42) is a sort of recurrent neural network that can learn long-term dependencies. An LSTM network typically comprises four layers: an input layer, two hidden layers, and one output layer. There are three gates in this system: forget gate, input gate, and output gate.

### 3.7. Evaluation metrics

The performance of these six classifiers is assessed using four metrics (43).

1. Accuracy: The number of accurately estimated sample points among all the sample points is known as accuracy. It is evaluated by using formula given in (1)
  (1) $$\begin{array}{r} Accuracy=\left(TP+TN\right)/\left(TP+FP+TN+FN\right)*100 \end{array}$$
2. Precision: It is the proportion of true positive cases to the overall number of illness cases. It is evaluated by using formula given in (2)
  (2) $$\begin{array}{r} Precision=TP/\left(TP+FN\right)*100 \end{array}$$
3. Recall: It's the proportion of true negative cases to the overall number of illness cases. And it is evaluated by using formula given in (3)
  (3) $$\begin{array}{r} Recall=TN/\left(TN+FP\right)*100 \end{array}$$
4. F1-Score: The F1-score is defined as the harmonic mean of the model's precision and recall and it is evaluated by using formula given in (4)
  (4) $$\begin{array}{r} F1-Score=2\;*\;\left(precision*recall\right)/\left(precision+recall\right) \end{array}$$

Where TP and TN denote the healthcare model's true positive and true negative predictions. The letters FP and FN stand for the healthcare model's false positive and false negative predictions.

## 4. Results and discussion

This section focuses into the results of classification algorithms, including SVM, logistic regression, ANN, CNN, RNN, and LSTM. The proposed model is implemented by using diabetes dataset obtained from the Kaggle data center, provided by the National Institute of Diabetes. The reason to choose these datasets is because the data is gathered from massive diabetes databases. These data come from a variety of pregnant women of different ages. The description of diabetes dataset is given in Table 1.

**Table 1:** Specifications of diabetes dataset.

**Item name**	**Normal**	**Low**	**Abnormal**	**Critical**
Pregnancies	3 = 5	1 = 3	6 = 9	=10 above
Glucose	70 mg/dL	110 mg/dL = 130	130 mg/dL = 160	=160 above
Blood pressure	80 mm Hg	80 mm Hg = 90	90 mm Hg = 100	=100 mm Hg
Skin thickness	25 = 30	0 = 25	3,040	40 = 50
Insulin	Fasting 25 ml U/L	30 min, 30–230 ml U/L	1 h 18–276 mlU/L	3 h,25 ml U/L
Body mass index	18.50 = 25	25 = 30	30 = 35	=35
Age	21–28	28–34	35–45	46–50

**Figure 2:**
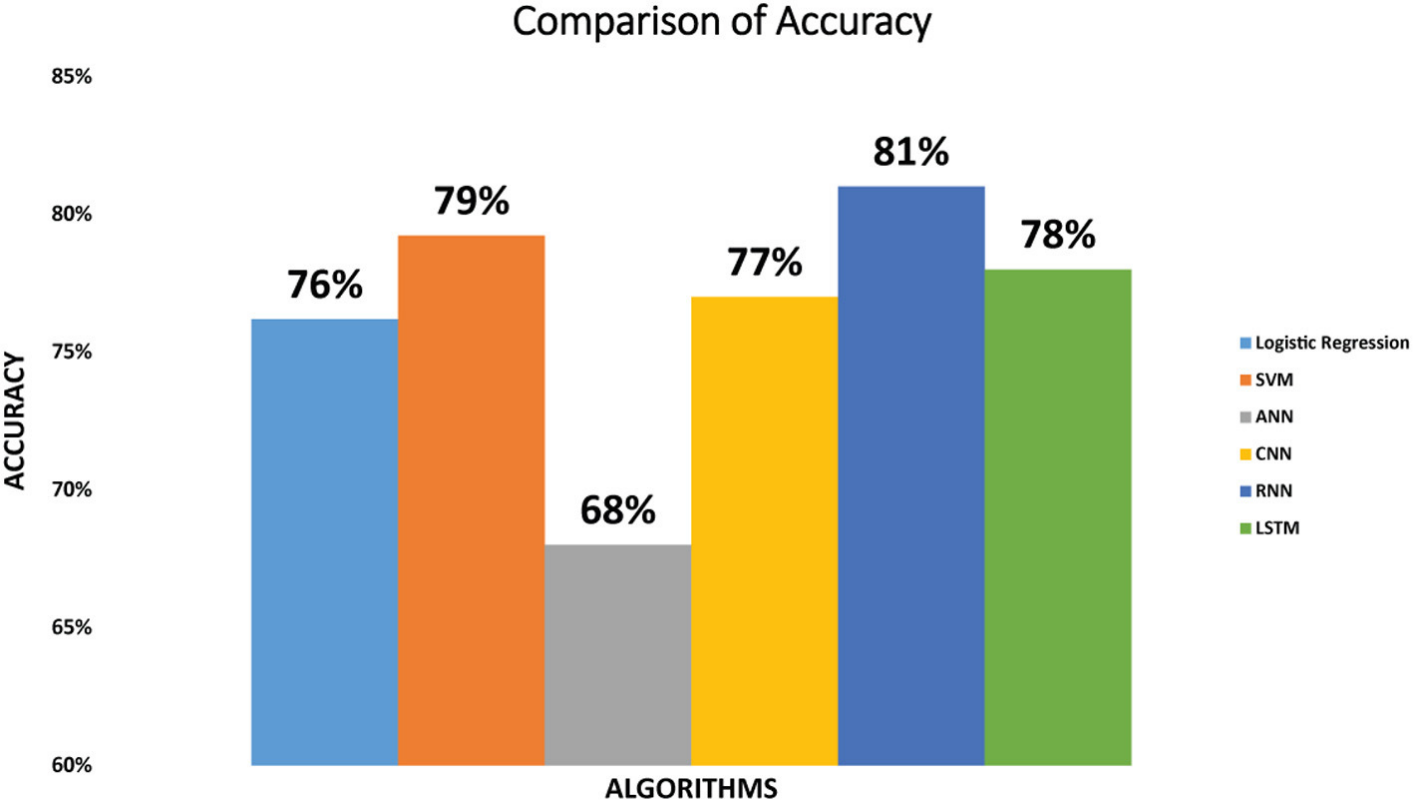
Comparison of accuracy for logistic regression, SVM, ANN, CNN, RNN, and LSTM.

Accuracy of the six classifiers: The accuracy we have achieved by implementing SVM classifier on diabetic dataset is 79%. Logistic regression attained the accuracy of 76% while the ANN classifier attained 68% accuracy. CNN achieved the accuracy of 77%. RNN achieved the accuracy of 81% and accuracy achieved by the LSTM is 78%. As we can see that RNN classifier achieved maximum accuracy as compared to the other five classifiers. The accuracy of the evaluated classifiers is shown in Figure 2.

**Figure 3:**
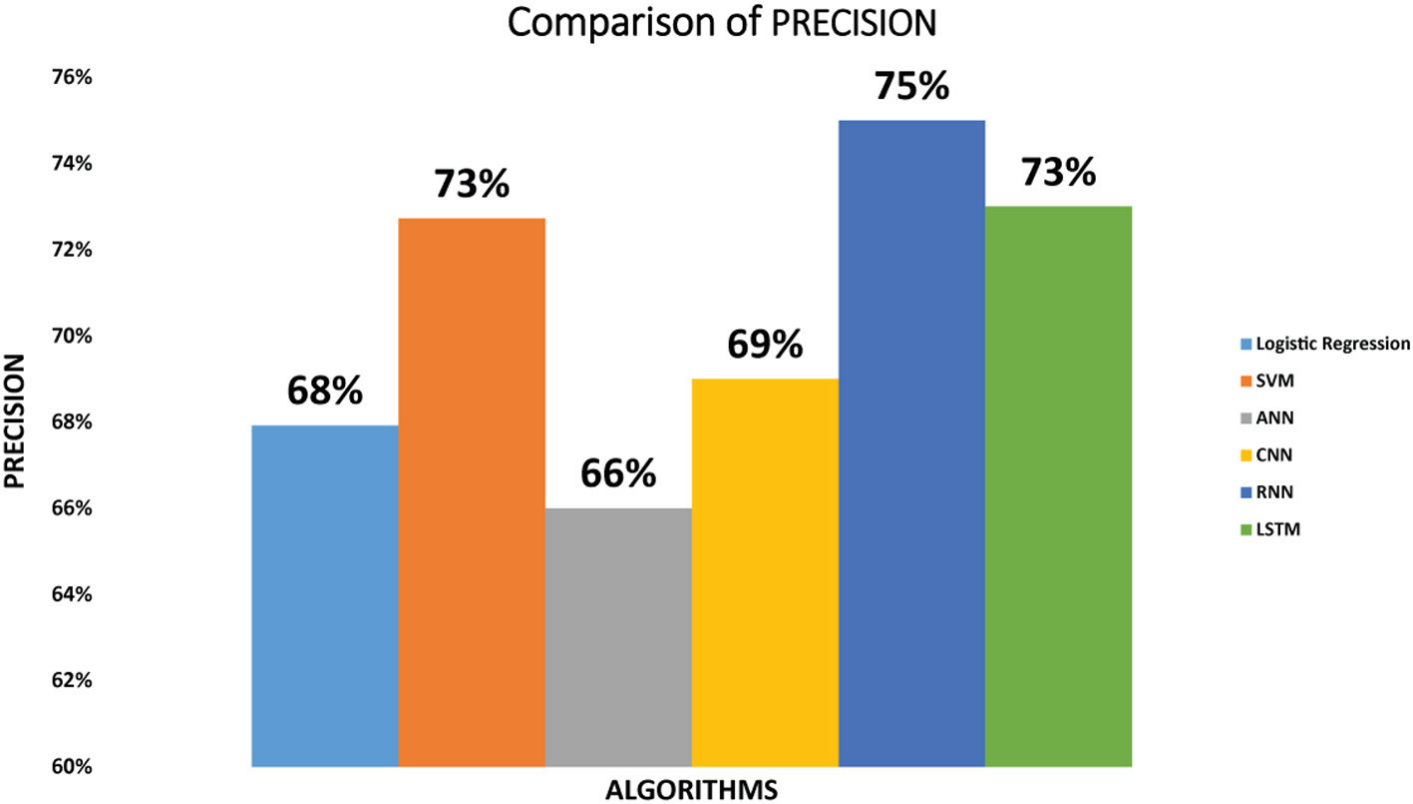
Comparison of precision for logistic regression, SVM, ANN, CNN, RNN, and LSTM.

Precision of the six classifiers: The precision we have achieved by implementing SVM classifier on diabetic dataset is 73%. Logistic regression attained the precision of 68% while the ANN classifier attained 66% precision. Precision of CNN is 69%. The precision achieved by RNN is 75% and the precision achieved by LSTM is 73%. As we can see that RNN classifier achieved maximum precision as compared to other classifiers. The precision of the classifiers is shown in Figure 3.

**Figure 4:**
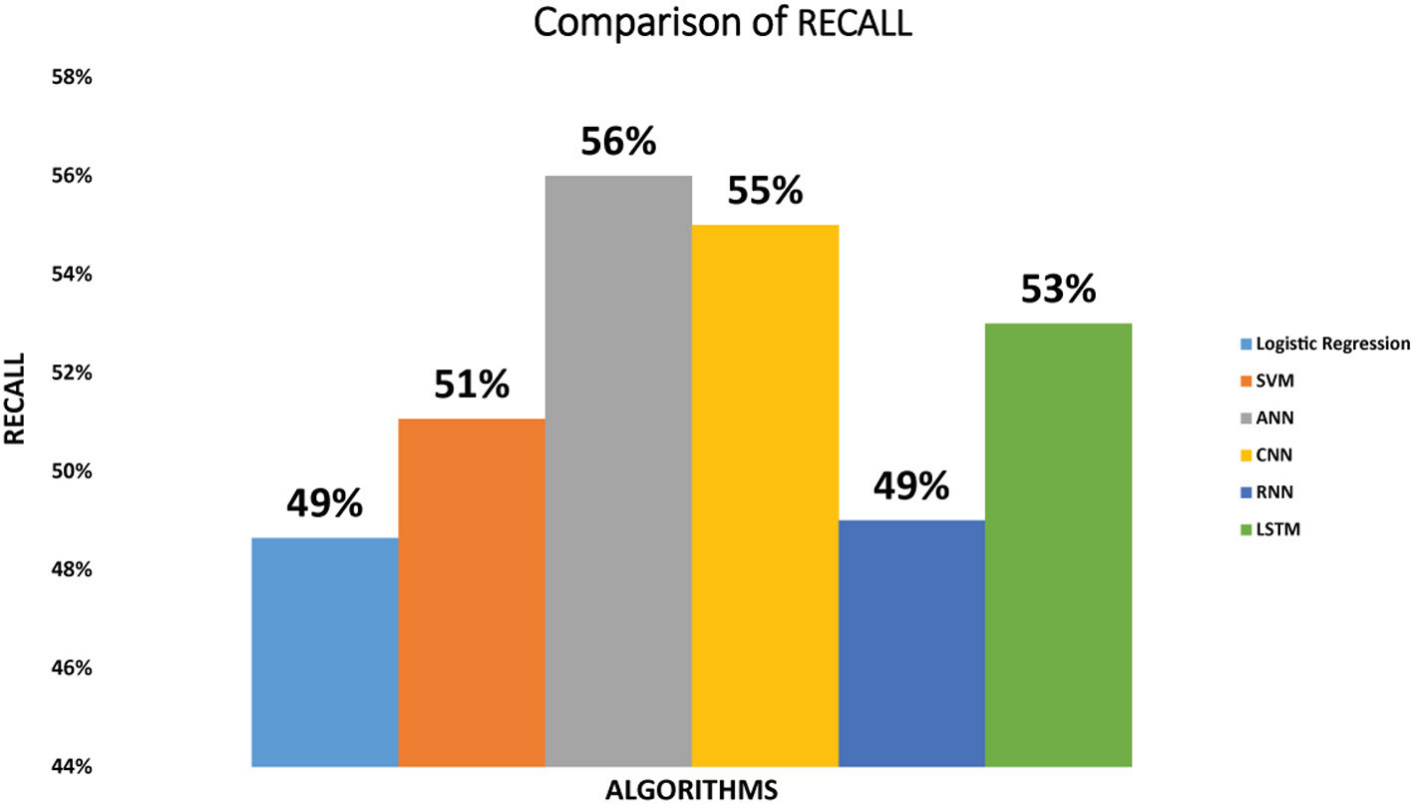
Comparison of recall for logistic regression, SVM, ANN, CNN, RNN, and LSTM.

Recall of the six classifiers: The recall we have achieved by implementing SVM classifier on diabetic dataset is 51%. Logistic regression attained the recall of 49% while the ANN classifier attained 56% recall. Recall achieved by CNN is 55%. RNN attained 49% recall and LSTM achieved Recall of 53%. As we can see that ANN classifier achieved maximum recall amongst other classifiers. Figure 4 depicts the recall achieved by all the classifiers.

**Figure 5:**
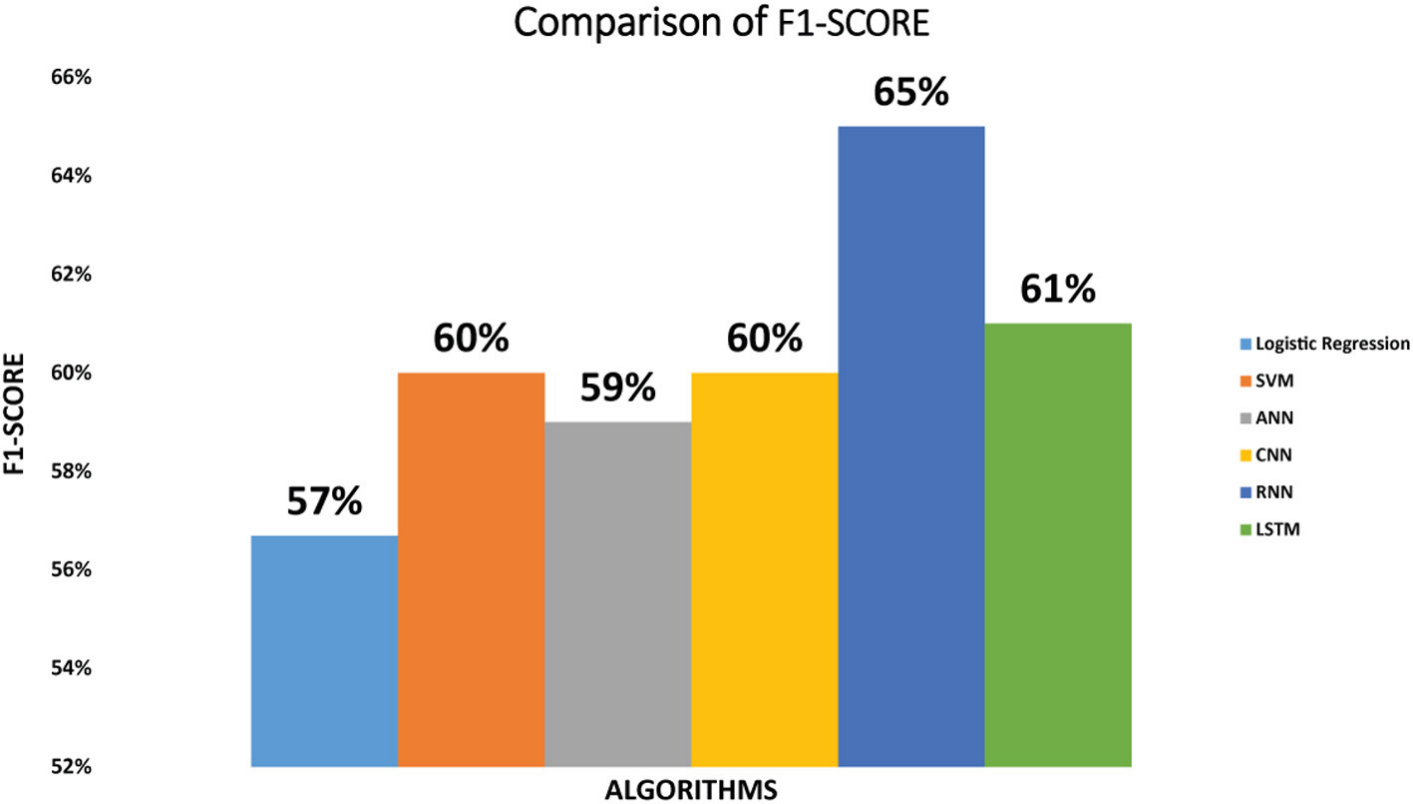
Comparison of F1-score for logistic regression, SVM, ANN, CNN, RNN, and LSTM.

F1-Score of the six classifiers: The F1-Score we have achieved by implementing SVM classifier on diabetic dataset is 60%. Logistic regression attained the F1-Score of 57% while the ANN classifier attained 59% F1-Score. CNN achieved 60% F1-Score. F1-Score achieved by RNN is 65% and LSTM obtained 61% F1-Score As we can see that RNN classifier achieved maximum F1-Score as compared to other classifiers. Figure 5 depicts the F1-Score achieved by all the classifiers.

**Figure 6:**
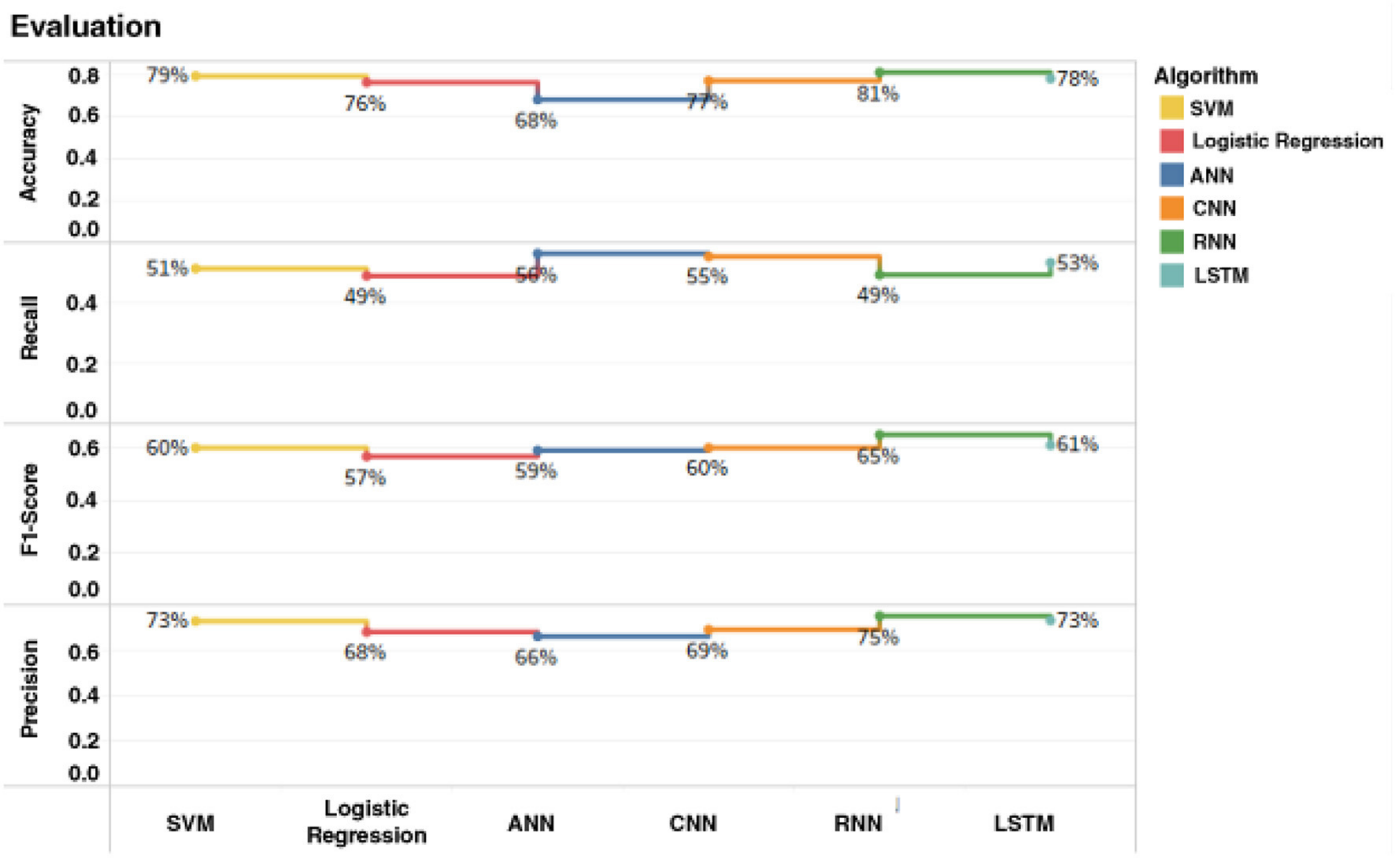
Comparable graphical view of accuracy, precision, recall, and F1-Score for the classification approaches i.e., SVM, Logistic regression, ANN, CNN, RNN, and LSTM.

Figure 6 represents the comparable graphical view of accuracy, precision, recall, and F1-Score for the classification approaches i.e., SVM, Logistic regression, ANN, CNN, RNN, and LSTM.

Accuracy, precision, recall, and F1-Score of the implemented classifiers is represented in Table 2.

**Table 2:** Performance evaluation of SVM, logistic regression, ANN, CNN, RNN, and LSTM for given dataset.

**Classifier**	**Accuracy**	**Precision**	**Recall**	**F-score**
	**(%)**	**(%)**	**(%)**	**(%)**
SVM	79	73	51	60
Logistic regression	76	68	49	57
ANN	68	66	56	59
CNN	77	69	55	60
RNN	81	75	49	65
LSTM	78	73	53	61

## 5. Conclusion

Machine learning and Internet of Things are the modern-day technologies that have the great capacity of revolutionizing the diabetes risk prediction owing to the advanced computational methods and availability of plethora of diabetes risk dataset. An important problem in the medical world is the early detection of Diabetes. In this research, we have proposed a smart patient's health monitoring system that uses the trending technologies such as Internet of Things and Machine learning to predict Diabetes at its early stage. We evaluated six machine learning algorithms i.e., support vector machine (SVM), logistic regression, artificial neural network (ANN), Convolutional Neural Network (CNN), recurrent neural network (RNN), and Long Short-Term Memory (LSTM). To investigate the robustness of each model, accuracy, precision, recall, and F1-score were calculated and compared with others. We performed the experiments on Pima Indian Diabetes dataset. Experimental results showed that RNN is the most ideal algorithm for predicting Diabetes, which gives an accuracy, precision, and F1-Score that are higher than remaining algorithms. However, the recall for ANN was higher as compared to SVM and logistic regression, CNN, RNN, and LSTM. As in this research we have focused on single demographic (pregnant females) dataset. As a future work we will implement these classifiers on a dataset that will focus on a broader audience.

## Data availability statement

Publicly available datasets were analyzed in this study. This data can be found here: https://www.kaggle.com/datasets/uciml/pima-indians-diabetes-database.

## Ethics statement

Ethical review and approval was not required for the study on human participants in accordance with the local legislation and institutional requirements. Written informed consent for participation was not required for this study in accordance with the national legislation and the institutional requirements.

## Author contributions

AN and RH conceived the model and project. RH implemented the learning models. TN, RH, SA, MAt, and MAr analyzed the results. AN, RH, and TN wrote the manuscript. All authors read, edited, and approved the final manuscript.

## Funding

This research was funded by Princess Nourah bint Abdulrahman University Researchers Supporting Project number (PNURSP2022R239), Princess Nourah bint Abdulrahman University, Riyadh, Saudi Arabia.

## Conflict of interest

The authors declare that the research was conducted in the absence of any commercial or financial relationships that could be construed as a potential conflict of interest.

## Publisher's note

All claims expressed in this article are solely those of the authors and do not necessarily represent those of their affiliated organizations, or those of the publisher, the editors and the reviewers. Any product that may be evaluated in this article, or claim that may be made by its manufacturer, is not guaranteed or endorsed by the publisher.
